# Trial Participation in Neurodegenerative Diseases: Barriers and Facilitators: A Systematic Review and Meta-Analysis

**DOI:** 10.1212/WNL.0000000000209503

**Published:** 2024-06-03

**Authors:** Daphne N. Weemering, Anita Beelen, Tessa Kliest, Lucie A.G. van Leeuwen, Leonard H. van den Berg, Ruben P.A. van Eijk

**Affiliations:** From the Department of Neurology (D.N.W., T.K., L.A.G.v.L., L.H.v.d.B., R.P.A.v.E.), Department of Rehabilitation, Physical Therapy Science & Sports (A.B.), and Center of Excellence for Rehabilitation Medicine (A.B.), UMC Utrecht Brain Center, University Medical Center Utrecht; De Hoogstraat Rehabilitation (A.B.), Utrecht; and Biostatistics & Research Support (R.P.A.v.E.), Julius Center for Health Sciences and Primary Care, University Medical Center Utrecht, the Netherlands.

## Abstract

**Background and Objectives:**

Clinical trials in neurodegenerative diseases often encounter selective enrollment and under-representation of certain patient populations. This delays drug development and substantially limits the generalizability of clinical trial results. To inform recruitment and retention strategies, and to better understand the generalizability of clinical trial populations, we investigated which factors drive participation.

**Methods:**

We reviewed the literature systematically to identify barriers to and facilitators of trial participation in 4 major neurodegenerative disease areas: Alzheimer disease, Parkinson disease, amyotrophic lateral sclerosis, and Huntington disease. Inclusion criteria included original research articles published in a peer-reviewed journal and evaluating barriers to and/or facilitators of participation in a clinical trial with a drug therapy (either symptomatic or disease-modifying). The Critical Appraisal Skills Program checklist for qualitative studies was used to assess and ensure the quality of the studies. Qualitative thematic analyses were employed to identify key enablers of trial participation. Subsequently, we pooled quantitative data of each enabler using meta-analytical models.

**Results:**

Overall, we identified 36 studies, enrolling a cumulative sample size of 5,269 patients, caregivers, and health care professionals. In total, the thematic analysis resulted in 31 unique enablers of trial participation; the key factors were patient-related (own health benefit and altruism), study-related (treatment and study burden), and health care professional-related (information availability and patient–physician relationship). When meta-analyzed across studies, responders reported that the reason to participate was mainly driven by (1) the relationship with clinical staff (70% of the respondents; 95% CI 53%–83%), (2) the availability of study information (67%, 95% CI 38%–87%), and (3) the use or absence of a placebo or sham-control arm (53% 95% CI 32%–72%). There was, however, significant heterogeneity between studies (all *p* < 0.001).

**Discussion:**

We have provided a comprehensive list of reasons why patients participate in clinical trials for neurodegenerative diseases. These results may help to increase participation rates, better inform patients, and facilitate patient-centric approaches, thereby potentially reducing selection mechanisms and improving generalizability of trial results.

## Introduction

Randomized clinical trials have been the gold standard for evaluating new disease-modifying therapies but present a particular challenge in neurodegenerative diseases.^1,2^ Besides our limited understanding of the underlying pathophysiologic mechanisms, and the clinical heterogeneity between patients,^1^ enrollment involves some unique challenges because of the debilitating nature of the diseases: trials require more time to complete and the costs incurred are higher than other therapeutic indications.^3^ These challenges are further exacerbated by barriers that hinder trial participation.

Participation rates in clinical trials have been estimated to be as low as 2%–8% of all patients living with a certain condition.^4^ Significantly, participation rates among ethnic minorities, older patients, and late-stage disease patients have been estimated to be even lower.^5-8^ Among patients who are aware of the option of participation and who are willing to participate, only a minor fraction is eligible,^9,10^ and even fewer patients will ultimately complete the study.^11^ This not only jeopardizes the generalizability of clinical trial results but also forms a major obstacle to feasibly executing a clinical trial and to developing a reliable understanding of the effectiveness and safety of a therapy afterward.^12^ Exploring the underlying mechanisms behind patient accrual, retention, and motivation to participate in clinical trials is, therefore, of significance.

Previous studies have focused on investigating barriers to and facilitators of trial participation in other therapeutic areas, including oncology, cardiology, stroke, and human immunodeficiency virus research.^4,13-19^ Numerous factors have been identified that influence trial participation. These encompass a range of considerations, such as personal motivations and factors related to the study design (e.g., placebo use, time consumption).^4,13^ However, because patients with neurodegenerative diseases often face a far more devastating future combined with functional or cognitive disabilities, factors that drive trial participation may differ. Therefore, we systematically reviewed the literature to identify factors that influence trial participation in 4 major neurodegenerative disease areas: Alzheimer disease (AD), amyotrophic lateral sclerosis (ALS), Huntington disease (HD), and Parkinson disease (PD). By combining a qualitative thematic analysis with a quantitative meta-analysis, we aim to provide a comprehensive overview of key factors that contribute to trial participation and thus inform investigators how to align their recruitment and retention strategies better.

## Methods

### Search Strategy

The objective of the systematic review was to search for all original research articles that aim to identify and/or evaluate barriers to and facilitators of clinical trial participation in patients with AD, ALS, HD, or PD. Studies were identified in the PubMed and Embase databases, as well as by screening reference lists from relevant reviews. The search was limited to the most common neurodegenerative diseases: AD, ALS, HD, and PD.^20,21^ Search terms included “barriers,” “facilitators,” “clinical trials,” “participation,” “Alzheimer's disease,” “amyotrophic lateral sclerosis,” “Huntington's disease,” “Parkinson's disease,” and their synonyms (eAppendix 1, all supplementary material is also available on GitHub). The search was discussed with an information specialist and was limited to qualitative and quantitative studies written in English. The final reference list was generated in May 2023.

### Study Selection

After removal of duplicates, the reference list was analyzed using Automated Systematic Review (ASReview)—an active (machine) learning framework that ranks titles and abstracts, based on the relevance of the record, using prior information entered into the software by the reviewer.^22^ For each disease group, 1 relevant record and 1 irrelevant record were selected to serve as prior information. During the review, ASReview presents an article to the reader based on prior information (i.e., which articles were relevant and which were irrelevant) and the reviewer decides whether the title and abstract fulfill the criteria, and thus, whether the article is relevant or irrelevant. Title and abstract reviewing with ASReview was halted when the number of consecutive irrelevant articles equaled 10% of the total.

Initially, a set of 200 ranked abstracts was created and screened by 2 reviewers. These were discussed until consensus was reached (eAppendix 2.1). The subsequent titles and abstracts were screened by 1 reviewer. References were eligible for full-text screening if the abstract described that the aim of the study was to identify and/or evaluate barriers to and/or facilitators of trial participation in clinical trials for neurodegenerative diseases. Full-text articles had to meet the following criteria to be used in the thematic analysis and meta-analysis (eAppendix 2.2): original research published in a peer-reviewed journal, evaluating barriers to and/or facilitators of participation in a clinical trial with a drug therapy (either symptomatic or disease modifying), and concerning patients with AD, ALS, HD, or PD. To assess and ensure the quality of the qualitative studies, the Critical Appraisal Skills Program (CASP) checklist for qualitative studies was used (eAppendix 3).^23^

### Data Extraction

Data on barriers and facilitators were independently extracted following a prespecified extraction scheme. The first 10 articles and the corresponding study extraction schemes were discussed in-depth and compared between 2 reviewers. Studies solely reporting demographics, people at risk of a neurodegenerative disease (e.g., healthy older patients), outcomes represented by <5% of the sample size, analyses without descriptive statistics, or articles where the patient population with neurodegenerative diseases was ≤50% of the total sample size were excluded from the analysis.

### Qualitative Synthesis

Thematic analysis was employed to integrate and interpret the qualitative data from the studies that were included. An inductive approach was used to determine the final themes. First, 2 researchers (T.K. and D.N.W.) independently coded the extracted data; then, the coding schemes were compared. The extracted data consisted of first-order constructs (i.e., direct patient quotes reported in the included studies) and second-order constructs (i.e., statements made by the authors of the included studies).^24^ The coded data represented the main idea or feeling that was expressed. After initial coding, all codes were collated and grouped. Finally, patterns were identified to create themes and subthemes based on the codes. As each theme can consist of both negative (barriers) and positive (facilitator) statements (e.g., for financial compensation, a positive statement would be that someone was glad they received compensation for their effort, whereas a negative statement would be that they would have appreciated some compensation for their time), we hereafter refer to them as “enablers” of trial participation.

The identified enablers were clustered into 3 overarching factors: patient-, study-, or HCP-related factors. The study- and HCP-related enablers were considered *modifiable* factors that can be altered before or during a clinical trial and could potentially benefit recruitment strategies. Patient-related enablers were appraised as motivators or beliefs—*intrinsic* factors that cannot be directly amended to improve participation in clinical trials but may facilitate better education of patients. To ensure trustworthiness of the analysis process and of the results, several consensus meetings were held with D.N.W., A.B., T.K., and R.P.A.v.E. to discuss the created codes, the grouping into patient-, study-, and HCP-related factors, and the final (sub)themes. For transparency, the review process is available on GitHub.

### Statistical Analysis

The study characteristics are summarized as frequency (percentage), with the thematic analysis being presented visually as a flowchart. The flowchart illustrates the distribution of data points for each enabler, reflecting the number of quotes or statements extracted from the included studies. The quantitative data, namely the proportion of responders reporting a certain enabler in each study, were synthesized using meta-analysis to obtain pooled effect size estimates across studies. A meta-analysis was conducted for each *modifiable* enabler separately. From each quantitative study, we calculated a proportion of responders reporting a certain enabler; these were subsequently logit transformed. Mixed-effects logistic models were fitted to account for the binomial structure of the data and to account for between-study heterogeneity.^25^ The weighting of the meta-analyses was indirectly affected by the number of information sources and the number of responses in that more information contributed to better estimation of the overall effect size. The pooled estimated percentages and their 95% CIs were reported for each *modifiable* enabler in a forest plot. We anticipated that the diversity in question formulation regarding trial participation would introduce heterogeneity. To quantify between-study heterogeneity, we report τ^2^ together with the *Q* and *I*^2^ statistics.

As an exploratory analysis, we evaluated whether study heterogeneity was driven by disease population, especially as AD data originate primarily from caregivers.^3^ As such, we added an indicator variable for AD vs non-AD as covariate in a meta-regressive model; the odds ratio and 95% CI were reported, together with the reduction in between-study heterogeneity. All quantitative analyses are performed in R (version 4.2.1).^26^ The “metafor” package was used for the meta-analyses.^27^ All data and code for this manuscript are available on GitHub.

### Standard Protocol Approvals, Registrations, and Patient Consents

As this study is a systematic review and did not involve direct participation of human participants, approval by an ethical committee was not applicable.

### Data Availability

The data used in both thematic analysis and meta-analysis are accessible through a GitHub repository (github.com/daphneweemering/trial-participation). This repository remains indefinitely accessible. In the event of data unavailability on GitHub, it can be obtained upon request.

## Results

**Figure 1:**
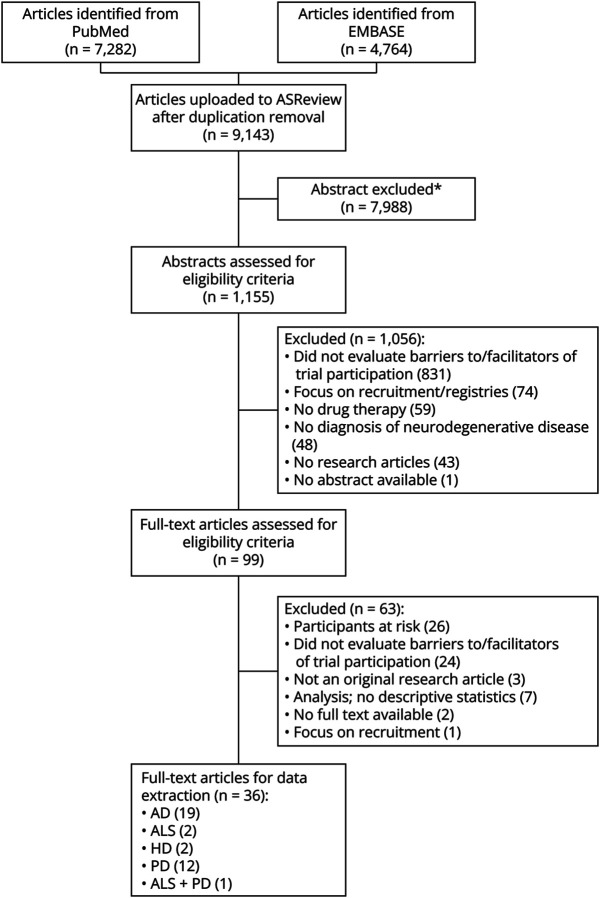
PRISMA Flowchart of Systematic Review Based on prior information (i.e., marking a relevant and an irrelevant article before title and abstract reviewing) and reviewer choices, the most relevant articles are pushed forward for review. Title and abstract reviewing was halted when the number of consecutive irrelevant articles equaled 10% of the total. *Abstracts are excluded based on ASReview systematic reviewing software. AD = Alzheimer disease; ALS = amyotrophic lateral sclerosis; ASReview = Automated Systematic Review; HD = Huntington disease; PD = Parkinson disease; PRISMA = Preferred Reporting Items for Systematic reviews and Meta-Analyses.

In total, 9,143 unique citations were identified; of these, 1,155 abstracts were screened in ASReview, and 99 full-text articles were included for the eligibility review (Figure 1). Inter-rater agreement was assessed for a fixed batch of 200 abstracts. Specifically, the kappa statistic (κ) was used to evaluate the degree of concordance between the reviewer assessments of the abstracts. This showed strong agreement (κ = 0.86) between 2 independent reviewers. Of the 99 articles assessed, 63 were excluded on the basis of predetermined criteria. Articles were excluded if they focused solely on patients at risk of a neurodegenerative disease (n = 26), lacked an assessment of barriers and/or facilitators of trial participation (n = 24), were not classified as an original research article (n = 3), performed an analysis and did not provide descriptive statistics (n = 7), were unavailable in full text (n = 2), or solely concentrated on recruitment (n = 1). An example of an excluded study is the study by Linger et al.,^28^ who evaluated the effect of several recruitment strategies on the likelihood of trial participation. No studies were excluded on the basis of the CASP, as the overall score for each study was adequate (eAppendix 3). Hence, 36 articles were used for data extraction and analysis (AD: n = 19, ALS: n = 2, HD: n = 2, PD: n = 12, ALS + PD: n = 1). The references to the included articles are given in eAppendix 4.

### Study Characteristics

Articles reporting different qualitative and/or quantitative methods, diverse responder types, or differing disease groups were treated as unique information sources, and data were extracted separately for each part of the article. As a result, the total number of unique information sources is greater than the number of articles. Overall, among the 36 articles, we identified 45 unique information sources enrolling a total sample size of 5,269 patients, caregivers, and HCPs, of which 18 sources were qualitative by design, 25 sources quantitative, and 2 sources combined qualitative and quantitative methods. The characteristics of the distinct information sources are summarized in Table 1; the characteristics of the individual studies are provided in the supplementary material (eAppendix 5). Of note, most information sources provided data from patients with either AD or PD (87%, 39 of 45). In addition, compared with the other disease areas, data for AD originated in 70% (16 of 23) from caregivers or patient–caregiver dyads; this was only 14% (3 of 22) for non-AD sources. For the non-AD sources, most data originated from patients.

**Table 1:** Summary of the Unique Information Sources

	AD (n = 23)	ALS (n = 4)	HD (n = 2)	PD (n = 16)	Overall (n = 45)
Total sample size	1,734	535	273	2,727	5,269
Data collection method					
Survey	12 (52)	4 (100)	1 (50)	8 (50)	25 (56)
(Semi)structured interview	7 (30)	—	1 (50)	4 (25)	12 (27)
Focus group	3 (13)	—	—	2 (13)	5 (11)
(Semi)structured interview and focus group	1 (4)	—	—	1 (6)	2 (4)
(Semi)structured interview, focus group, and survey	—	—	—	1 (6)	1 (2)
Responder type					
Patients	5 (22)	3 (75)	1 (50)	11 (69)	20 (44)
Patients and caregivers	7 (30)	—	1 (50)	1 (6)	9 (20)
Caregivers	9 (39)	—	—	1 (6)	10 (22)
Patients, caregivers, HCPs	—	—	—	2 (13)	2 (4)
HCPs	2 (9)	1 (25)	—	1 (6)	4 (9)

Abbreviations: AD = Alzheimer disease; ALS = amyotrophic lateral sclerosis; HCP = health care professional; HD = Huntington disease; PD = Parkinson disease.

For the data collection method and responder type, data are N (% of total information sources in disease group). This table includes the number of stand-alone information sources, not the number of articles. Articles combining qualitative and/or quantitative data collection methods, diverse responder types, or disease groups were analyzed separately.

### Emerged Enablers

**Figure 2:**
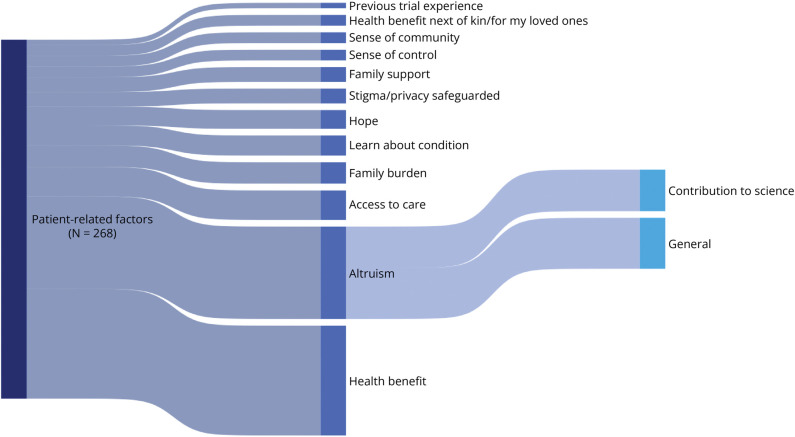
Weighted Flowchart of the Identified Patient-Related Enablers This figure depicts the identified patient-related enablers. The vertical blocks indicate the (sub)themes and enablers. The thickness of the “flows,” that is, the curved/smooth bars between the (sub)themes, is proportional to the number of data points for that (sub)theme. Hence, thicker flows indicate a higher frequency. The sample size (N) indicates the total number of data points.

**Figure 3:**
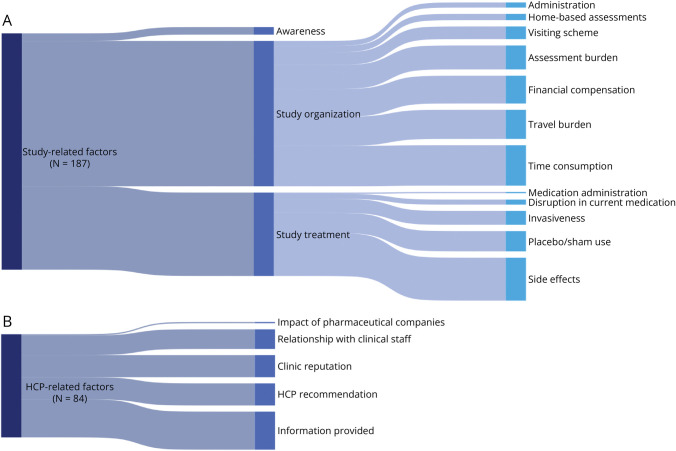
Weighted Flowchart of the Modifiable Enablers: Study and HCP Related This figure depicts the identified study- (A) and HCP-related (B) enablers. The vertical blocks indicate the (sub)themes and enablers. The thickness of the “flows,” that is, the curved bars between the (sub)themes, is proportional to the number of data points for that (sub)theme. Hence, thicker flows indicate a higher frequency. The sample size (N) indicates the total number of data points. HCP = health care professional.

**Figure 4:**
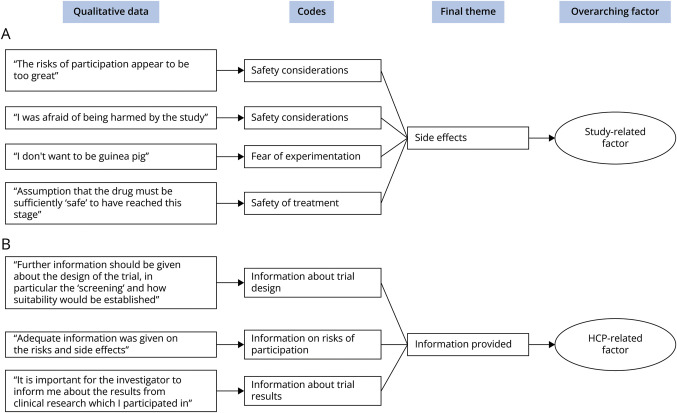
Illustrative Example of the Thematic Analysis Process In this figure, we illustrate the coding process of the enablers “side effects” (A) and “information provided” (B). Initially, data were collected from the included studies. Subsequently, an initial coding phase was undertaken. These codes were then organized into final themes. In the last step, the themes were grouped into patient-, study-, or HCP-related factors. HCP = health care professional.

A total of 539 qualitative data points (i.e., text segments including quotes and statements from the qualitative studies) were extracted from the enrolled study population. Of these data points, 268 (50%) concerned patient-related enablers, 187 (35%) concerned study-related enablers, and 84 (16%) concerned HCP-related enablers. These data could be deduced to 31 unique enablers, which are depicted in dynamic flowcharts. Figure 2 displays the identified patient-related enablers; Figure 3 displays the *modifiable* study- and HCP-related enablers. A thicker “flow” indicates a higher frequency of data allocated to a certain enabler. Enablers standing out in terms of frequency are personal health benefit (15% of total number of qualitative data points), general altruism (7%), side effects (6%), time consumption (6%), contribution to science (6%), and information provided by HCPs (6%). The complete code tree with qualitative data and frequencies is available in the GitHub repository. An illustrative example of the coding process is shown in Figure 4.

### Quantitative Synthesis

**Figure 5:**
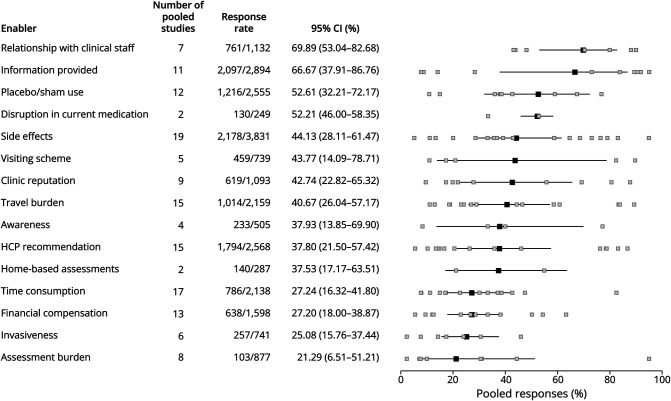
Forest Plot of Meta-Analyzed Reported Proportions of the Modifiable Enablers of Trial Participation The black boxes indicate the overall estimate of the generalized linear mixed-effects models with 95% CIs based on the Gaussian distribution. The gray boxes indicate the response rate of the individual studies. Articles including different responders, responder types, disease groups, or data collection methods were separately included in the analysis. HCP = health care professional.

Quantitative data on the *modifiable* enablers were reported in 27 of the 45 information sources (25 quantitatively designed sources and 2 qualitative sources reporting frequencies); a meta-analysis was conducted for each of these enablers (Figure 5). Overall, the relationship with the clinical staff was the preeminent enabler reported by 70% of the respondents (95% CI 53%–83%), followed by the availability of study information (67%, 95% CI 38%–87%) and the use or absence of a placebo or sham-control arm (53%, 95% CI 32%–72%). The disruption or continuation of usual care was also reported frequently among respondents (52%, 95% CI 46%–58%) but was only based on 2 studies primarily enrolling patients with PD.

Of note, for all enablers, there was considerable between-study heterogeneity (*I*^2^ ranging from 84% to 99%, all *p* < 0.001, eAppendix 6), likely because of the divergent types of survey methods used and the enrolled survey respondents (Table 1). Funnel plots of the 4 enablers with the largest pooled effect sizes (and where the number of information sources is greater than 2) also show large variation in the reported proportions and the precision of these proportions (eAppendix 7). In an attempt to explain the between-study heterogeneity, we evaluated whether responses were different for AD vs non-AD studies (Table 2). Significant differences between the AD responders and not-AD responders were observed for the relationship with the clinical staff (odds ratio [OR] 0.2, 95% CI 0.1–0.3), the visiting scheme (OR 0.1, 95% CI 0.0–0.6), and HCP recommendation (OR 0.1, 95% CI 0.0–0.4), which could explain 48%–90% of the between-study heterogeneity, although significant heterogeneity remained (eAppendix 6).

**Table 2:** Comparison of AD Studies (1) and Non-AD Studies (0)

Barrier/facilitator	Estimated percentage (95% CI)		Odds ratio	*p* Value
	Non-AD	AD		
Relationship with clinical staff	82.72 (76.72–87.43)	45.86 (35.32–56.79)	0.18 (0.10–0.32)	<0.01
Information provided	76.74 (45.59–92.86)	45.17 (11.51–83.92)	0.25 (0.03–2.49)	0.24
Placebo/sham use	60.69 (36.59–80.51)	35.53 (11.88–69.27)	0.36 (0.06–1.99)	0.24
Disruption in current medication^a^	—	—	—	—
Side effects	56.38 (35.73–75.04)	28.11 (12.37–52.01)	0.30 (0.08–1.14)	0.08
Visiting scheme	69.20 (36.49–89.78)	13.44 (2.74–46.15)	0.07 (0.01–0.61)	0.02
Clinic reputation	52.65 (28.49–75.63)	23.81 (6.21–59.62)	0.28 (0.04–1.81)	0.18
Travel burden	50.65 (30.12–70.97)	30.19 (14.58–52.28)	0.42 (0.12–1.50)	0.18
Awareness	37.08 (10.69–74.38)	39.97 (5.42–88.55)	1.13 (0.06–21.06)	0.93
HCP recommendation	59.22 (40.83–75.35)	14.37 (6.08–30.29)	0.12 (0.03–0.39)	<0.01
Home-based assessments^a^	—	—	—	—
Time consumption	38.72 (22.14–58.41)	17.10 (7.91–33.12)	0.33 (0.10–1.07)	0.06
Financial compensation	29.87 (19.05–43.53)	20.46 (8.44–41.81)	0.60 (0.19–1.97)	0.40
Invasiveness	19.27 (8.88–36.89)	29.41 (18.03–44.11)	1.75 (0.62–4.89)	0.29
Assessment burden	37.07 (9.08–77.65)	11.05 (2.08–42.10)	0.21 (0.02–2.58)	0.22

Abbreviations: AD = Alzheimer disease; HCP = health care professional.

Estimates are based on a generalized linear mixed-effects model with AD responder as moderator (1 = AD, 0 = non-AD).

a These enablers only have 2 separate studies, rendering the meta-regression uninformative.

## Discussion

The primary objective was to systematically identify factors that influence trial participation in neurodegenerative diseases and provide a comprehensive synthesis of the literature. We have shown the considerable diversity in enablers that drive patient participation. Using thematic deduction, key themes were identified that could be attributed to the patient, the study design, or the health care professional (HCP). Although intrinsic patient factors, such as altruism or personal health benefit, may be less directly modifiable, we identified several study and HCP-related factors that may increase the patient's likelihood of trial participation. These results could help to improve recruitment strategies to include a broader range of patients and to better educate and inform patients about participating in future clinical trials.

Understanding why patients do or do not participate in clinical trials is, however, not only relevant to improving accrual rates among clinical trials. It also provides a better understanding of the drug's safety and efficacy profile, and how applicable study results are to all patients with a certain disease.^29^ It is well known that only a minor fraction of the patients are eligible to participate in clinical trials for neurogenerative disorders.^9,10^ However, even among eligible patients, only a subset finally decide to participate.^11^ With eligibility criteria, the aim is to reduce undesirable characteristics in our trial population (e.g., end-stage disease). There may, however, be a second latent selection mechanism that determines whether an eligible patient will participate. This latent mechanism may be driven by another factor (e.g., age). As a result, our actual trial population deviates considerably from our intended one, which could make eligibility criteria ineffective.^10^

In a recent population-based study in patients with ALS,^30^ it was shown that of the 473 patients predicted to have been eligible according to the inclusion and exclusion criteria, only 133 (28%) finally participated in the study. Perhaps unsurprisingly, the patients who actually participated were different from the eligible nonparticipants. In our study, we provide a comprehensive list of factors that may drive these decisions and thus influence these latent selection mechanisms. Indeed, intrinsic patient-related factors play a major role, with altruistic reasons and own health benefit being major themes. Although these motivations are opposites, they are not mutually exclusive; many individuals may be driven by a combination of altruistic and self-interested factors. The observed discrepancy between eligible patients and actual participants highlights the complexity of the decision-making process surrounding trial participation. Our study underscores the importance of addressing barriers and misconceptions about the purpose, risks, and benefits of participation in a clinical trial. Development of better educational materials and consent procedures that match all educational levels and diverse needs may alleviate potential barriers to participate.^31^ Strategies such as interactive, informed consent interventions, such as a test with feedback in addition to a standard informed consent procedure, have been shown to be effective in improving patients' understanding of the implications of trial participation.^32^ Informing patients may be further improved by involving patient advocates or communication specialists.^33^

In addition, we show the significant influence of HCP-related factors on the patient's decision to participate in a clinical trial. Specifically, the relationship that patients and caregivers have with the clinical staff, ensuring that the patients understand the study design and that they receive trial results after participation, emerged as the most influential and potentially modifiable contributors to trial participation. These findings are supported by previous studies, further demonstrating that some patients may not fully comprehend the informed consent procedure, especially when it comes to understanding safety, side effects, and randomization.^34^ This is significant, as it influences the willingness to participate in a clinical trial,^35^ and patients may participate not being fully aware of the study purpose and risks associated.^15^ Furthermore, interventions aimed at addressing recruitment barriers have shown promise. For instance, in a clinical trial evaluating an exercise regimen, various recruitment strategies were assessed, with referrals by neurologists and primary care providers proving most effective.^36^ This underscores the role of HCPs at all stages of trial engagement.

Gathering the patient's input on the study design may be another pathway to promote trial participation.^37,38^ Patient-centered trial design has gained increasing attention in recent years and has garnered support from regulatory authorities.^39,40^ Our list of enablers offers valuable insights into which elements of study design are deemed important by patients, supported by other studies,^41,42^ including the use of placebo, the intensity of the visiting scheme, the travel burden, and the required time investment. Making better use of innovative trial designs that facilitate these patient preferences may well further enhance trial participation, for example, by making more frequent use of platform studies,^43-46^ interim analyses,^47^ hybrid designs to reduce placebo arms,^30^ or by providing a decentralized visiting scheme.^48^

Our study has several limitations that should be considered. First, the publications analyzed in this study were not specific to individual clinical trials, but rather examined overarching factors influencing trial participation. Given the diverse scope of neurodegenerative clinical trials and the varied focuses of the publications analyzed, it would be a challenge to estimate the proportion of all performed trials represented in the sample of included studies. The overall insights, however, could be valuable as guideline for future studies. Second, the diseases included in our study vary in their symptoms, caregiver involvement, and life expectancy. We have assessed the between-study heterogeneity for each enabler, with a clear difference between AD and non-AD studies, especially in the enablers' “relationship with clinical staff” and “visiting scheme.” This may not be surprising, given that most AD studies were based on the response of caregivers. Third, the quantitative studies assessed only a limited set of enablers relative to the set of enablers identified in the qualitative studies. Hence, subsequent quantitative studies can be improved by incorporating a more comprehensive set of enablers based on qualitative studies, following a standardized study design and survey format. Another avenue of interest may be to explore methodologies such as conjoint analyses to learn how alterations in attributes of a clinical trial (design) improve willingness of patients to participate.^49^ For instance, a study involving patients with AD and their caregivers employed conjoint analysis to assess which trial design factors would enhance participation. Study findings revealed that factors such as the burden of traveling to the clinic were important barriers, with home visits emerging as most valued alternative to in-clinic follow-up.^49^ This example underscores the potential value of tailored trial designs, based on patient input, to address specific participation barriers identified in our study.

Finally, it would be of specific interest to do future analyses in populations that are difficult to enroll, such as ethnic minorities, older patients, and those with a lower socioeconomic status.^50^ Although some studies in this review focused on factors that drive or hinder trial participation in under-represented populations, sample sizes were too limited to identify differential factors influencing trial participation in neurodegenerative diseases. However, given that under-representation of these subgroups in clinical trials profoundly affects the trial's generalizability, efforts are needed to gather specific insights tailored to under-represented subpopulations.

In conclusion, we have identified and quantified the considerable diversity in enablers that drive patient participation in clinical trials for neurodegenerative diseases. Understanding these enablers is critical for designing effective recruitment strategies and optimizing the conduct of clinical trials. These results could help to improve recruitment and retention strategies and be applied to better educate and inform patients about participating in future clinical trials, ultimately advancing the development of effective treatments for neurodegenerative diseases.

## Appendix Authors

**Table:**

Name	Location	Contribution
Daphne N. Weemering, MSc	Department of Neurology, UMC Utrecht Brain Center, University Medical Center Utrecht, the Netherlands	Drafting/revision of the manuscript for content, including medical writing for content; major role in the acquisition of data; study concept or design; analysis or interpretation of data
Anita Beelen, PhD	Department of Rehabilitation, Physical Therapy Science & Sports, and Center of Excellence for Rehabilitation Medicine, UMC Utrecht Brain Center, University Medical Center Utrecht; De Hoogstraat Rehabilitation, Utrecht, the Netherlands	Drafting/revision of the manuscript for content, including medical writing for content; analysis or interpretation of data
Tessa Kliest, MSc	Department of Neurology, UMC Utrecht Brain Center, University Medical Center Utrecht, the Netherlands	Major role in the acquisition of data; study concept or design
Lucie A.G. van Leeuwen, PhD	Department of Neurology, UMC Utrecht Brain Center, University Medical Center Utrecht, the Netherlands	Major role in the acquisition of data
Leonard H. van den Berg, MD, PhD	Department of Neurology, UMC Utrecht Brain Center, University Medical Center Utrecht, the Netherlands	Drafting/revision of the manuscript for content, including medical writing for content
Ruben P.A. van Eijk, MD, PhD	Department of Neurology, UMC Utrecht Brain Center, and Biostatistics & Research Support, Julius Center for Health Sciences and Primary Care, University Medical Center Utrecht, the Netherlands	Drafting/revision of the manuscript for content, including medical writing for content; study concept or design; analysis or interpretation of data

## Glossary

AD: Alzheimer disease
ALS: amyotrophic lateral sclerosis
ASReview: Automated Systematic Review
CASP: Critical Appraisal Skills Program
HCP: health care professional
HD: Huntington disease
OR: odds ratio
PD: Parkinson disease
